# “*I think it is quite naive to think everybody’s goal is that*”: how Zambian sexual violence stakeholder perspectives complicate global health roadmaps to ‘decolonization’

**DOI:** 10.1186/s12913-025-13188-5

**Published:** 2025-10-15

**Authors:** Nancy Nyutsem Breton, Nancy Lwimba Mukupa, Mazuba Mushota-Mafwenko

**Affiliations:** 1 Department of Methodology, London School of Economics and Political Science, London, England, UK; 2 University of Zambia, Lusaka, Zambia; 3 University of Missouri, Columbia, MO 65211 USA

**Keywords:** Decolonization, Sexual violence, SGBV, Zambia, Global health, Roadmap, Community empowerment

## Abstract

**Background:**

The global health and development field is embracing calls to decolonize, producing ‘roadmaps’ to decolonial practices. These calls are echoed in the field of sexual and gender-based violence (SGBV), where entrenched global structural power relations undermine the potential for the community-centered, liberatory change to which decolonial roadmaps aspire. Despite the ubiquity of such calls, empirical research on the prospects for their implementation remains limited. This paper investigates the readiness among SGBV-related institutions in Zambia to address coloniality. We ask: can a decolonial praxis be realized amidst entrenched barriers, and is the global health and development industry ready to implement roadmaps for decolonization?

**Methods:**

We conducted 19 interviews with Zambia-based donor, implementing agency, and grassroots stakeholders involved in SGBV policy and programs. We performed a critical thematic analysis to explore the complexities within the country's SGBV interventions.

**Results:**

While the Zambian Anti-Gender-Based Violence Act and subsequent policies aimed to transform the SGBV landscape by establishing a systemized approach, we find considerable discrepancies between intervention expectations and the local implementation realities. Norms contributing to SGBV, a perception of a “bad” Zambian culture, and conflicting social values impede the impact of legal instruments. These challenges not only hinder sustainable implementation of transformative policies, but also reflect deeper structural and epistemic inequities that undermine efforts to pursue a decolonial praxis. As such, they illuminate how colonial legacies continue to shape policy and intervention outcomes and constrain the feasibility of decolonization in practice.

**Conclusion:**

This paper argues that addressing power dynamics and profit-driven motives is crucial for genuine transformation and will require a recalibration of current systems. The disparity between these factors raises critical questions about decolonization and the potential for alternative, community-focused interventions which give people agency over their liberation. The current study speaks to broader global health and development discussions by spotlighting the challenges to transformative interventions in Global Majority contexts. Confronting these challenges is essential for reshaping narratives around the implementation of decolonial roadmaps and the aspiration to community empowerment.

**Supplementary Information:**

The online version contains supplementary material available at 10.1186/s12913-025-13188-5.

## Introduction

Recent calls to decolonize global health and development offer powerful critiques of the hierarchies embedded in international aid and knowledge production. Scholars, activists, and institutions alike have advocated for a reimagining of interventions and policies, urging a shift towards more equitable and contextually grounded approaches [1]. A recent scoping review of colonialism and decolonization in violence against women and girls (VAWG) research and programming underscores this growing reckoning, highlighting widespread calls to center community knowledge, interrogate donor-driven priorities, and resist the dilution of decolonial ideals into rhetorical buzzwords [2]. Entities such as The Lancet [3], the London School of Hygiene and Tropical Medicine’s Health in Humanitarian Crises Centre (HHCC) [4], AmplifyChange [5], the Hewlett Foundation [6], and Ipas [7] have produced research and committed to decolonial strategies. To move from theory to practice, organizations and scholars have produced ‘roadmaps’ in the form of actionable strategies and guidelines. Simultaneously, substantial financial resources within the same sector target sexual- and gender-based violence (SGBV) [8], underscoring the importance of addressing SGBV within the broader decolonization narrative.

Through a global mental health lens, Burgess ([9], p. 49) critiques how colonization and its legacies contribute to the medicalization of global health and development, neglecting structural determinants in favor of individualized solutions. Such approaches reveal intangible amplifications of power and trauma, perpetuating neglect of transformative, community-centered solutions. This tendency resonates with concerns in SGBV discourse, where external framing within the global health paradigm risks overshadowing critical empowerment aspects ([9], p. 27). Examining the tangible manifestations of power and trauma in SGBV helps surface those intangible residues from colonial legacies. This paper draws on interviews with SGBV stakeholders in Zambia to explore how everyday power dynamics—alongside structural constraints, epistemic dominance, and institutional logics—complicate idealistic decolonial roadmaps in global health and development.

### Mapping decolonization: navigating global health perspectives

Scholars and practitioners offer diverse interpretations of decolonizing global health—from reformist measures centered on organizational acknowledgment and trackable progress [10], to more radical approaches reshaping leadership and knowledge frameworks [11] and requiring a complete systemic overhaul [12]. We align with these more radical articulations, defining decolonization as a process of centering marginalized communities, dismantling systemic inequities, and promoting collective accountability. This includes recognizing survival pressures and institutional constraints that shape how Global Majority communities navigate and reimagine existing systems. In this paper, we examine prospects for such radical decolonization in the context of SGBV interventions in Zambia.

Various existing initiatives [13], including those addressing global sexual and reproductive health and rights (SRHR) issues, advocate strategies such as localizing power and resources [14], bolstering research anchored in Global Majority countries [5], restructuring power imbalances [15], and fostering accountability and structural change [16] to embody a decolonial praxis within institutional efforts. Specifically, a WHO framework [17], centered on enhancing institutional engagement with people affected by non-communicable diseases (NCDs) and mental health conditions, emphasizes adopting participatory approaches that address structural and systemic inequities, striving to realign power structures with decolonialization efforts. The framework promotes “critical ‘allyship,’” rooted in principles of “antiracism, anti-oppression, anti-colonialism, [and] anti-discrimination…with a rights-based, pro-equity approach to engagement” ([17], p. 32). While these commitments to decolonize are noble, they might be daunting for those grappling with the realization that the industry they passionately serve is fundamentally flawed. This prompts the question: What concrete measures can be enacted to facilitate decolonization?

A proposed three-step roadmap by Khan and colleagues [10] outlines crucial actions, focusing on organizational acknowledgment, reforms, and measurable progress tracking. However, it does not explicitly address key foundational concepts such as white supremacy, western exceptionalism, racism, ableism, homophobia, transphobia, and capitalism that are inherent in colonial legacies. This limitation restricts collective accountability and structural liberation. In contrast, the Global Health Decolonization Movement in Africa (GHDM-Africa), confronts discomfort by explicitly naming coloniality and its sociopolitical and economic underpinnings. Their 2021 guide [18] provides actionable guidance for change, for instance, by advocating inclusive leadership teams and institutional restructuring to restore decision-making power to Global Majority communities, rectifying the systemic perpetuation of coloniality in global health.

While we support GHDM-Africa’s approach, we offer a complementary framework that questions the assumption that all institutions and communities are equally prepared to adopt decolonial methods. Drawing on interviews with stakeholders engaged in Zambia-based SGBV interventions, we examine how decolonial aspirations are shaped and constrained by operational realities. Stakeholders'reflections reveal how coloniality persists within institutional structures, often in subtle but consequential ways. Understanding these dynamics requires critical attention to power, history, and local context. By focusing on Zambia, we illustrate how systemic barriers complicate efforts to implement context-sensitive and empowering approaches, raising broader questions about the feasibility of decolonial praxis in the field.

### Barriers to decolonization in global health and development

Reviewing how SGBV is addressed within a Global Majority country reveals three significant limitations that hinder the potential for decolonial praxis in the context of global health and development. First, SGBV interventions themselves reinforce colonial, hegemonic, and misogynistic relations. Through a one-dimensional framing [19], women are presented as choice-disabled victims who need to report more [20–23] and men as ignorant and choice-disabled perpetrators threatened by economic imbalances [20, 23–25]. These narratives perpetuate power imbalances, positioning international organizations as saviors [26] needed to"civilize"or"liberate"these communities. To implement a genuinely decolonial praxis, intervention discourses that echo those from colonial regimes must be explored, questioned, and dismantled.

Second, monitoring, evaluation, and research practices contribute to problematic knowledge production within global health and development organizations. Exemplified by initiatives like MEASURE Evaluation [27], a project whose indicators focus primarily on technical aspects and individual behaviors, these approaches sidestep the broader sociohistorical context underlying SGBV. This narrow perspective implies that addressing technical inputs alone suffices, overlooking the systemic and structural roots of the issue [28]. Scholars, like Piedalue and colleagues [29], advocate for comprehensive mixed methods approaches grounded in feminist critiques of power hierarchies to interrogate the complex influences of SGBV.

Third, intervention models often overlook the practical challenges faced on the ground, as evidenced by the implementation gaps in one-stop centers (OSCs) for intimate partner violence (IPV) survivors in low- and middle-income countries [30]. Despite interventions intending to streamline post-violence care, real-world obstacles, including limited resources and poor institutional collaboration, divert attention from addressing the root causes. This diversion perpetuates a standardized framework that exclusively values empirical evidence as the ultimate solution, neglecting the diverse local contexts and values at play. As Chigudu ([31], p. 1875) aptly argues, it “attempts to make the world in its own image and thus rides roughshod over the experiences, knowledge, and values” of local communities.

These limitations are relevant in the Zambian context, as we explore below. As the interviews with SGBV institutional stakeholders demonstrate, these challenges manifest within a Zambian institutional framework in ways that echo the critiques above.

### Unveiling Zambia’s contextual intersections

Studying Zambia offers an opportunity to delve into the intricate complexities within and surrounding decolonization efforts in Global Majority countries. Exploring how Zambian SGBV practitioners perceive and navigate intervention challenges offers grounded insight into the operational and systemic barriers that constrain transformative change in global health and development.

Zambia is a compelling case study due to its high SGBV prevalence: Specifically, 47% of Zambian women and girls aged 15–49 reported experiences of IPV in the last 12 months [32], while 16.9% of young women aged 15–24 experienced non-IPV during the same period ([33], p. 7). These figures reflect broader trends across the Southern Africa Development Community (SADC), where IPV prevalence ranges from 25–58% [32]. National efforts to mitigate SGBV are ongoing—informed by the 2011 Anti-Gender-Based Violence (Anti-GBV) Act [34], However, other perspectives have criticized the limitations of the community impact of the Act [33]. Examining the complexities of Zambia’s initiatives elucidates the intricate interplay between institutional structures, colonial legacies, and the potential to operationalize decolonial principles within the context of global health and development.

Complementing Zambia’s legislative efforts, various donor-funded initiatives have sought to strengthen prevention and response systems in alignment with the Anti-GBV Act [35]. For example, the UN Joint Program on Gender Based Violence, the EU-funded NATWAMPANE program [36], and the USAID PEPFAR-funded Stop Gender-Based Violence (STOP GBV) project [37] all focus on community mobilization, norms change, and enhancing local system capacity. These initiatives employ tactics such as one-stop centers (OSCs), fast-track courts, victim support units (VSUs), engagement with traditional leaders, and the provision of protective shelters.

Organizational evaluations and scholarly analyses of these programs and the Act itself reveal that, while Zambia’s efforts to address SGBV represent a significant step forward [38, 39], there are problems with how it has been problematized and collaboratively addressed [40]. Further, the existing legal frameworks often exhibit contradictions between policies [41], and limited funding, resource capacity, accessibility, and geographical reach collectively hinder the effective realization of policies and interventions [42]. Chungu [43] critiques the lack of community centering and impact of existing efforts, emphasizing the need for individual and collective accountability to challenge the social attitudes and norms that perpetuate such behaviors.

The commendable legislative and programmatic strides made in Zambia around SGBV, together with their persistent challenges, are the foundation of this paper. We lay the groundwork necessary for exploring the interplay between legislative efforts and their implementation, before examining how they interact with the broader sociocultural context. This context grounds our subsequent analysis of systemic barriers and their implications for effective SGBV interventions and decolonization efforts in global health and development.

Zambia’s shift from post-independence nationalism to neoliberalism continues to shape its approach to SGBV and global health. Following independence in 1964, the country struggled with the legacy of colonial resource exploitation and debt [44]. President Kaunda’s rejection of International Monetary Fund (IMF) austerity measures in 1987 briefly interrupted this trajectory [45], but economic pressures—including inflation, fluctuating copper prices, and debt burden—led to renewed neoliberal reforms under President Chiluba and the Movement for Multiparty Democracy (MMD) in 1991 [45].

Following Harden [46], we argue that the re-adoption of neoliberal policies and agreements has led to a relentless cycle of financial pressures for Global Majority countries. Chiluba’s assertion that Zambia would emulate “successful capitalist economies in the West,” [45] underscores the strategic alignment of neoliberalism with global financial institutions, potentially perpetuating the cycle of dependence. In a context where even the current Zambian president has signed on to an anti-poor IMF agreement to reduce the national fiscal deficit [47], how might it be possible for local-level stakeholders to envision alternative approaches beyond neoliberal mechanisms to effectively address social inequity?

This paper contributes to global health and development literature by examining how the systemic and operational dynamics of SGBV interventions shape the feasibility of decolonial praxis — an area that remains underexplored. While grounded in the Zambian context, the insights offered speak to broader challenges confronting Global Majority countries navigating donor influence, entrenched institutional norms, and resource constraints. Through stakeholder interviews, we illuminate how coloniality persists within the institutional practices of global health, and how this constrains more transformative, community-rooted approaches. These findings underscore the need to move beyond rhetorical commitments to decolonization and address the power structures embedded within everyday development practice.

## Methodology

### Zambia as a case study

Zambia’s sociopolitical history, as previously outlined, provides context to its relevance when problematizing the tensions between global decolonial aspirations and localized challenges in addressing SGBV and wider global health and development issues.

This historical trajectory reflects a cycle of operational pressures for Global Majority countries and their potential relationship with systemic issues. Kaunda's move highlighted the dilemma of balancing national development with the burden of debt repayment [46], stemming from colonial resource exploitation. Zambia's subsequent return to IFI agreements emphasized alignment with global financial institutions, potentially perpetuating dependency [45]. Even recent agreements, such as the IMF deal to reduce the fiscal deficit [47], underscore mainstream economic approaches that may not fully address structural concerns. This historical context resonates with our research question, exploring the intricate interplay between decolonial aspirations and institutional realities that impact and are influenced by efforts to address SGBV in the context of global health and development. Zambia is an illuminating case study due to its representative struggles with the operational pressures faced by Global Majority countries, reflecting broader patterns of post-colonial recovery and neocolonial practices. Its historical trajectory contextualizes how systemic issues intersect with local challenges, providing insights into the complexities of addressing SGBV within the global health and development framework.

### Research design & approach

The co-authors constituted a collaborative international team, committed to a dialogical and decolonial approach to qualitative research, aiming to offer an expansive use of qualitative methods to transcend silos and transform perspectives [48]. Self-identifying as Black African women, with one in the diaspora and two as Black Zambians, all three of us are professional qualitative global health and development evaluators, with experience spanning Zambia, western Africa, and the United States. These identities contextualize our perspectives on the project and data interpretation. We collaboratively recruited participants, conducted interviews, and performed a thematic analysis on the data.

At the project's onset, our understandings of what decolonization meant for Zambia-based SGBV efforts varied. Through iterative discussion and shared reflection, we arrived at a more collective and nuanced grasp of its meanings and challenges. We embraced these internal tensions as productive and aligned with our decolonial commitment to hold space for complexity. Our approach values the friction of divergent views as a resource for collaborative structural transformation.

### Data collection

We conducted nineteen in-depth interviews with institutional stakeholders working on Zambia-based SGBV efforts, spanning donor agencies, international and local implementing organizations, and grassroots groups (see Table 1 for affiliations). We sought to understand how stakeholders viewed intervention design, success, collaboration, policy gaps, community engagement, and the relevance and feasibility of decolonization in the global health and development context.

**Table 1:** List of nineteen (19) interviewees by sector affiliation

Sector Affiliation	# of Participants
Donor	3
International Implementing Agency	5
Local/Zambian Implementing Agency	8
Grassroots Organization	3

Participants were identified through informal professional networks, internet searches, and snowball sampling to ensure broad representation. Interviews were conducted remotely—via phone, Zoom, or Skype—between July 2020 and March 2021, using a semi-structured guide developed collaboratively by the authors ([49], see Supplementary Material).

### Ethical considerations

The ethics considerations for this study encompass several key facets. First, anonymity was upheld to safeguard the identities of participants, particularly in critiquing stakeholders with good intentions. This measure ensures that individuals are not unduly scrutinized or portrayed negatively, thereby encouraging actors to reflect constructively on their actions and contributions.

Second, ethical sampling methods were employed to represent a diverse array of voices integral to the SGBV discourse. This intentional inclusivity resonates with the principles of decolonization, prioritizing the perspectives of historically excluded voices, including grassroots organizations, local implementers, and community members directly affected by SGBV. By embracing these perspectives, the study not only upholds ethical standards for equitable dialogue but also enriches interventions by grounding them in the local context, enhancing their efficacy and relevance.

Further, adherence to principles of informed consent was paramount throughout the research process. Participants were fully informed about the nature of the study, its objectives, and potential implications, allowing them to make voluntary and informed decisions about their involvement.

### Analysis

Analysis was informed by Lawless and Chen’s [50] expansion of Owen’s thematic analysis approach. Embracing critical thematic analysis, we examine identified themes through a lens of social justice [50], pp. 96–97). This aligns with our aim to critically scrutinize barriers impeding the implementation of decolonial practices within global health and development.

**Fig. 1:**
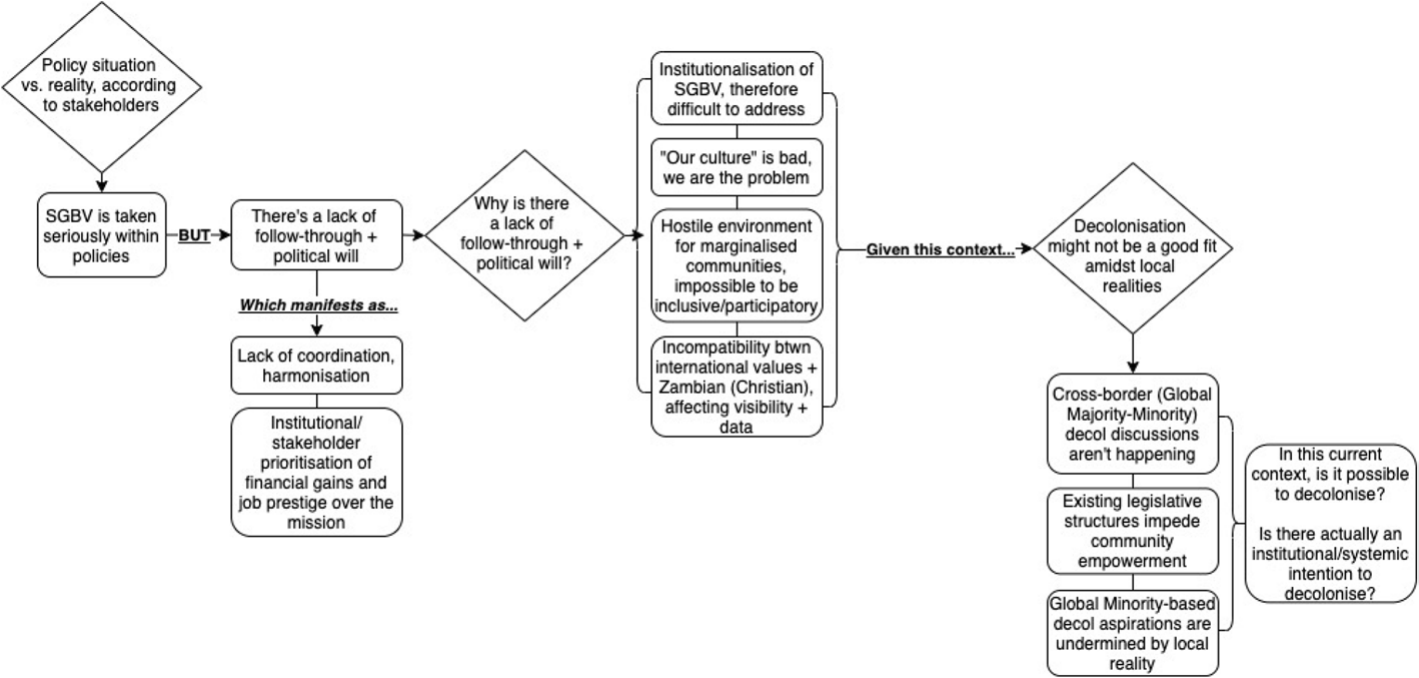
Flow diagram of themes contextualizing decolonial aspirations in current policy and practice

We used Dedoose software because its cloud-based nature facilitated international synchronous collaboration. After each independently coding the first transcript, we compared our coding for patterns, themes, and areas of convergence. Through these conversations, we discovered a considerable degree of similarity between our coding choices and resolved some instances of conflicting codes. Ultimately, we opted to retain some divergences, as they represented our pluralistic perspectives and the inherent nuances present in the data. We then coded all the transcripts, transferring coded excerpts into Excel to detect emerging themes. These themes were reviewed by each co-author and synthesized by the lead author into a flow diagram to illuminate the processes undermining decolonial aspirations (Fig. 1).

## Findings

Here, we outline three critical aspects of stakeholder perspectives on SGBV interventions in Zambia and their implications for global health and development, with particular attention to how systemic and operational barriers shape and constrain aspirations toward a decolonial praxis. First, we examine the gap between policy intentions and practical implementation. Second, we explore the lack of policy follow-through and political will, identified by stakeholders, impacting effective action. Lastly, building on these tensions, we consider nuanced tensions surrounding decolonization within global health and development. These findings complicate research and practical endeavors aimed at operationalizing a decolonial praxis. They also show that what might seem like general implementation challenges are actually shaped by deeper structural conditions—legacies of colonialism that make decolonial transformation difficult within current institutional systems.

It is important to acknowledge and celebrate organizational achievements, especially in a field grappling with such serious and complex issues. Addressing SGBV in a collaborative and sustainable manner is challenging, given the structural, societal, and emotional nuances surrounding violence and social inequity. Key stakeholders are tirelessly doing their best with the available resources. Bearing this in mind, several interviewees shared notable accomplishments of existing SGBV efforts. These include fast-track courts that reduce retraumatizing delays have contributed to a reduction in SGBV case withdrawals, multi-country and cross-sector collaborations that improve referrals and support providing SGBV survivors with both material and healing support, and increased initiatives engaging traditional leaders to raise SGBV awareness and empower community members to take a stand against violence. These accomplishments reflect tireless efforts amidst resource constraints.

### Policy situation vs. reality, according to stakeholders

One of the biggest achievements noted by stakeholders was the implementation of policies and laws, such as the 2011 Anti-GBV Act and the 2014 National Gender Policy, which they report have been instrumental in creating a systematized response to SGBV. These policies were seen as innovative and foundational, reflecting a positive societal shift by contributing to increased public dialogue and community support. This underscores the significance of robust implementation in realizing the potential of these policies.

While the policies serve as crucial foundations and establish a framework for designing and implementing SGBV interventions, gaps in implementation persist. Some respondents emphasized how specific policies, such as the Anti-GBV Act, work in tandem with others to precisely define and address SGBV. However, others highlighted inconsistent coordination between laws and the challenges posed by the time-consuming and politically sensitive process required for amendments or repeals. One stakeholder from a local implementing agency stressed that legal inconsistencies within the Constitution create ongoing challenges for the outlined legal reforms from the Anti-GBV Act, complicating the realization of otherwise promising policies.

Other stakeholders echoed similar views, pointing to insufficient political will as a contributing factor to the gap between policy guidelines and tangible outcomes. This highlights a critical issue where policy intentions often struggle to materialize into concrete actions and results.

“In my experience there hasn’t been anything harmonized. Again, it seems to link to that lack of just drive [...] I don’t get frustration, I just get a lack of passion. I can tell these people were passionate at some time, definitely, and they still care but they’re tired [...] I feel like people don’t necessarily walk the talk. Everybody talks. Yes, it’s wrong. Yeah, we need to do something. But then when you see like how much money, how much resources they put behind it—and this is everybody—then you see a huge difference between what they say and what they actually do.” (Donor)

“The more people get to the top the more people are in the space of they are making money and they are making a name for themselves. Suddenly, they are not doing the work anymore.” (Grassroots organization)

The above quotes reflect a shared sentiment about the disparity between expressed commitment and tangible efforts. Both donor and grassroots stakeholders recognize that despite high levels of initial commitment, with more experience or advancement to more senior positions, a sense of demoralization or apathy gradually sets in. Money and resources were another common concern—whether in how funding is allocated to SGBV efforts, or in how institutional actors’ income shape their long-term engagement. Stakeholders viewed these two factors as significant contributors to the observed lack of SGBV policy implementation, which is echoed by other respondents:

“One of the challenges is the lack of resources and information sharing among the service providers. The issue of different ministries having different resources. For instance, Ministry of Health is big, they have a lot of resources compared to Ministry of Home Affairs [the Police] and some local NGOs. This makes coordination a problem. For instance, if you want to respond to a case of gender-based violence, the Zambia Police has no vehicle, but the Ministry of Health has a vehicle. The Ministry of Health cannot release a vehicle for Zambia Police to go and respond to the issue of GBV, so that becomes a challenge. Also, Zambia Police has a vehicle but yet no resources for fuel but Ministry of Health or the Judiciary department has fuel, but they will not help Zambia Police with fuel.” (Local implementing agency)

This stakeholder highlights the relationship between political will, inequitable access of resources, and the lack of coordination between institutions. Overall, stakeholders offered mixed views on institutional coordination. Some saw collaboration and joint planning as promising (with some shortcomings), while others expressed skepticism, citing poor harmonization and information sharing. Their perspectives reinforce the disconnect between policy intentions and practical realities in SGBV interventions.

“It depends. The civil society or NGOs always work hand in hand because they understand each other, and they have a common goal. But when it comes to the Ministries, because they have policies that they have to abide to, you find there is a bit kind of conflicts. For example, even if they know that this is wrong because they have to follow certain policies, they won't come out as the real them. They will come out as what's on the paper. But when they see us, they always say, ‘At least you should speak for us because you know us, we need to protect our jobs.’” (International implementing agency)

These implementation failures are not merely operational oversights—they reflect enduring colonial governance legacies where hierarchical control, fragmented accountability, and externally driven priorities continue to shape how policy is enacted on the ground. Such systemic dysfunction undermines the very foundations required for a genuine decolonial shift.

### Why is there a lack of follow-through and political will?

Institutional survival and self-preservation emerged as key concerns. The international implementing agency stakeholder, above, noted that supporting intersectional SGBV efforts could cost actors their jobs—reflecting how transformative efforts are constrained by internal hierarchies. This echoes earlier concerns about political will and helps explain the lack of consistent policy follow-through. These insights point to how policy implementation is shaped not just by institutional priorities, but by the survival logics of individuals within them. Several stakeholders described the institutionalization of SGBV as a barrier in itself, suggesting that the very systems tasked with addressing violence may inadvertently reproduce the conditions that sustain it.

These observations suggest that institutional failures stem not only from capacity constraints but also from entrenched logics of control, exclusion, and hierarchy that are structurally resistant to the redistributive and power-sharing aims of decolonization. The institutionalization of SGBV mirrors colonial tendencies to manage and depoliticize violence rather than address its root causes.

Several stakeholders emphasized the need for cultural transformation, pointing to entrenched patriarchal norms and systemic gender inequalities that permeate both institutions and broader society. While some acknowledged problematic cultural norms, they also noted the importance of challenging these under the prevention framework. Collectively, these views reveal how culture and institutional practices interact to sustain SGBV.

“Well, definitely we have entrenched patriarchal tendencies, entrenched systemic approach to the way society views women and girls. We have a way in which women and girls have held a low status in society, and we have those inequalities across gender [...] So all those things make GBV thrive [...] It is just common knowledge wherever one may go whether it’s in the parliament, a cooperate institution, or in a school.” (Donor)

In emphasizing the need for a cultural transformation, two stakeholders from local implementing agencies highlight how deeply embedded social norms obstruct efforts to implement SGBV policies effectively. Others provide further context, illustrating the influence of “bad” culture across all social hierarchies in the community, noting that survivors often remain silent out of fear of further marginalization. These fears are particularly acute among LGBTQ + individuals, who face legal criminalization and social exclusion.

This fear of further marginalization not only discourages reporting but also obscures the scope of SGBV, as many affected groups remain invisible in official data. A grassroots stakeholder described how queer Zambian women face disproportionate sexual violence and systemic neglect within their communities. Even if these survivors do make informal disclosures, the data cannot be shared externally, preventing the development of responsive, multi-pronged SGBV interventions:

“ [...] if I look at the target groups that we are looking at—the female sex workers, men having sex with men, and the transgender persons—when they encounter SGBV [...] they usually refrain from reporting it [...because, usually,] the medical practitioner will want a police report and, once they go to the police, by virtue of their sexual orientation or sexual identity they will probably be denied a police report or they will be stigmatized, and sometimes even criminalized [...] as a result, one of the greatest challenges we have faced as a project is we have very little data because a lot of these cases are reported but they will not allow us to document them.” (International implementing agency)

This data suppression reflects a broader pattern of state-sanctioned erasure, underpinned by the hegemonic perception that queer communities are incompatible with Zambian cultural values:

“When we talk about every person should be treated in a decent manner, every person is equal before the law, whether you're gay, you're lesbian, you're what, whether you're straight, whether you are Christian, whether you're an Atheist [...] before the law you're the same. This nonsense of saying that no, we we're a Christian nation so we don't want gay people here, who wants you as a Christian here? We don't want them [...] And if someone is saying that person doesn't belong here, we don't want such people in Zambia.” (Local implementing agency)

### Is decolonization a good fit amidst local realities?

The State-sanctioned intolerance and invisibility directed at these communities directly contradicts the inclusive and progressive rhetoric of relevant policies and laws. Entrenched social norms and institutional silences cast doubt on the viability of a truly decolonial approach. Stakeholder reflections raise difficult but necessary questions: given persistent challenges like poor policy implementation, resource scarcity, and financialized priorities, is decolonization a viable or even relevant framework in this context? Or does the global health and development industry’s push for decolonial roadmaps risk overlooking the structural and epistemic work still required on the ground?

These questions are not isolated, but rather emerge directly from the lived realities and frustrations voiced by stakeholders. Through these interviews, stakeholders have highlighted the systemic and operational barriers pervading SGBV efforts. These findings illustrate that challenges in SGBV policy implementation are not isolated technical failures, but structural manifestations of donor-driven and colonial legacies. In this sense, the very barriers to effective implementation also represent core impediments to a decolonial agenda. As stakeholders strive to address or navigate these challenges, they grapple with the fact that they are operating within a framework of policies and standards that do not align with their community context. Stakeholders acknowledge this disjuncture, describing the lack of localization as a central impediment to effective practice:

“I don’t think certain policies from western countries can apply because we are living in different worlds. So, some they can work, some they can't apply because they are developed for that context and for us our culture has a strong influence. So, we need to have policies that suit our context.” (International implementing agency)

Although some may argue that decolonization can be advanced through collaborative partnerships between international actors and local communities, stakeholders shared that such conversations are largely absent. A donor representative admitted that decoloniality is not discussed:

“[...] we don’t talk about [decolonization] at all [...and] that’s an area certainly that is an issue and certainly should be addressed. But it’s not anything that has been discussed since I’ve been gender focal point. And, I mean, it’s so much broader than just SGBV or global health, right? I mean it’s just general governance.” (Donor)

And, even where decolonial ideas might gain traction, legal frameworks continue to limit community participation and impact:

“In terms of policy, this is a huge monster or the big elephant in that we have [...] government for the people by the people and policy reform must be participatory [...] But in reality, I personally feel that this does not necessarily happen [...] at the end of the day when these documents are being documented, some of these key elements are left out because of what is being guided at the Constitution level.” (International implementing agency)

The fact that decolonization is not being discussed on the ground, coupled with the challenge of establishing participatory mechanisms, reflects the persistence of historical power dynamics that limit community agency and empowerment.

“[...] we have all these groups, we have all these conversations on GBV, we have all these things, but to some point it’s all about the conferences [...] There are people who I have met who are doing amazing work who you see and you learn from and they are just too little. They do not have the sort of power, it’s now very political. People who only care about GBV when it’s Women’s Day. They care about it when it’s time for elections, but nothing, not even our government [...]” (Grassroots organization)

This lack of dialogue around decolonization, particularly one inclusive of voices with less power, may contribute to a limited understanding as to what decolonization is meant to address and achieve. Furthermore, the perception of policy directives as disconnected from the local context, coupled with frustration towards the modus operandi of SGBV efforts and the enduring systemic and operational barriers, provides context for the resistance among some stakeholders towards considering a decolonial praxis, or even envisioning its relevance and feasibility. For example, one stakeholder questioned the relevance of decolonizing SGBV, advocating instead for universal human rights frameworks:

“[...] this is a human rights issue. It has nothing to do with me being Black or white, whether I am here in Zambia or in Europe. This transcends race or color, greed or whatever you can call it. And we cannot say that our approaches to it are because of the things we are adopting from the developed world. These are basically human rights issues [...] Therefore, it has nothing to with a foreign concept. I am actually struggling to understand people’s perspective of decolonizing it.” (Local implementing agency)

Other stakeholders welcomed decolonial thinking—when locally contextualized—but raised concerns about continued reliance on colonial language and systems, with some viewing decolonization as a challenging and unfamiliar process. These tensions highlight a core dilemma: how can a decolonial praxis genuinely take shape in a setting where both the tools and discourses for transformation remain misaligned with community realities?

Stakeholders from grassroots organizations, as well as local and international implementing agencies stress the importance of acknowledging and addressing SGBV as an indigenous issue, highlighting the need to integrate the local context rather than adopting solutions from the Western world. They express concerns about the ongoing dependency on external funding, highlighting the challenge of balancing the desire to decolonize with the practical realities of resource limitations and the influence of donor priorities. Ultimately, these interviews encourage us to think about the sincerity behind the global health and development industry’s aspirations to decolonize. As the industry forges ahead in crafting interventions and publishing roadmaps for decolonization, it becomes evident through the lens of these interviews that without addressing the entrenched systemic and operational barriers confronting communities, institutional claims to decolonize may remain surface-level gestures rather than catalysts for meaningful change.

“I think it is quite naive to think everybody’s goal is [decolonization]. Really. And you got to look way above the issue of SGBV and [...] what is the goal of international development [...] Is it power dynamics or is it to make sure everybody you know this leave no one behind what not [?...] And I think that’s where we need to start to be looking at. We could say one thing, but really what is most important to them?” (Donor)

The themes explored above illuminate the complexity of stakeholder experiences navigating Zambia’s SGBV landscape—from policy-practice gaps and limited political will to the deeper colonial logics embedded in global health structures. Figure 1 presents a visual synthesis of these findings, mapping how systemic and operational barriers cascade across multiple levels to obstruct progress toward a decolonial praxis, even as stakeholders express both aspiration and ambivalence about its possibilities.

## Discussion

Stakeholders consistently described a disconnect between SGBV policy aims and their real-world implementation. While many valued efforts, such as the 2011 Anti-GBV Act and the 2014 National Gender Policy, they pointed to persistent challenges that undermine impact—including resource constraints, poor coordination, prioritization of financial gains, misallocation of funds, entrenched social norms, and institutionalization of SGBV. While these barriers are often framed as technical or logistical, our findings suggest that they are structural manifestations of a global health and development industry that continues to reproduce colonial power relations. These systemic issues do not merely constrain implementation; they expose the conditions under which decolonization is rendered nearly impossible. The lack of consistent policy follow-through and political will emerges as a critical point of friction — less so because of inefficiency, because of embedded interests and hierarchies resistant to transformation.

### Poor coordination and harmonization between policies and institutions

Participants noted that while the Anti-GBV Act identifies SGBV forms, it lacks specified penalties, leading organizations to rely on the Penal Code. Though initial perceptions suggest a disparity in policy harmonization, closer examination reveals a reliance on carceral measures and conventional justice. The current framework, shaped by hegemonic structures, constrains alternative reimagination, hindering transformative, community-centric approaches aligned with decolonial aspirations. This discordance requires a critical re-evaluation of justice paradigms within established systems.

Surajpal [51] acknowledges flaws in pre-colonial African justice systems and highlights the colonial imposition of prisons to align with Western ideals (51, p. 7). Prior to colonization, various African communities utilized reparative and transformative justice systems, alongside retribution. Today’s carceral responses mirror colonial influence by displacing those traditions in favor of punitive systems. This displacement not only reproduces the systemic marginalization of communities but also obstructs the potential to address SGBV through indigenous, community-rooted practices. Given that SGBV itself reflects power imbalances [52–54], confronting carceral responses is essential for any meaningful decolonial praxis.

Carcerality, rooted in colonialism and sustained by neoliberal logics [55, 56], reflects how global health and development continues to favor solutions that are measurable, fundable, and institutionally palatable over those that are transformative and emancipatory. This presence encourages critical reflections on the industry's genuine commitment to a decolonial agenda. Surajpal [51] argues that true decolonization requires an epistemological shift that rejects the Global Minority's imposed reform on Global Majority countries and, instead, recovers the humanitarian, rehabilitative, and community foundational ideals of various African nations as a starting point to envision what could emerge. Redressing imbalances requires a multifaceted approach, involving communities, addressing root causes, providing comprehensive support, and promoting survivor-centric, transformative, and community-based solutions. If global health and development seriously desires to push the industry towards decolonization, engaging in decarceration with local stakeholders and communities might be essential to initiate the necessary shift [57, 58].

### Prioritization of financial gains and prestige over the imperative to address SGBV

Stakeholders'reflections on inadequate harmonization and collaboration often revealed the influence of profit-driven motives and prestige-seeking behaviors within the industry. While some described policy advocacy successes, yet also noted how bureaucratic processes and donor logics shifted focus from community wellbeing to institutional survival.

These accounts highlight a broader critique of how global health and development sustains a form of economic colonialism that maintains dependence on external aid while reinforcing power imbalances that benefit privileged actors. The perception that some stakeholders are motivated more by financial gain or prestige than community wellbeing reveals the risk of commodifying issues like SGBV—treating them not as matters of justice, but as economic opportunities. Eyben’s [59] substantiates this critique through an examination of efforts to align care work with existing economic paradigms by revealing how such attempts reinforce hegemonic ideologies within international development. Addressing SGBV should involve providing critical support and services to survivors, demanding a compassionate and empathetic response to those who have experienced trauma. In essence, these stakeholder perspectives illuminate how interests and power dynamics are woven into the fabric of global health and development, manifesting as symbolic empowerment that falls short of addressing trauma, inequity, and exclusion. Their accounts offer compelling evidence of a system struggling with the commodification of crucial issues, thus making it challenging to navigate towards decolonial approaches. This complexity is further exacerbated by the industry’s prevailing norm of under-resourcing and funds misallocation.

### Limited resources and misallocation of funds towards SGBV efforts

The identified resource limitations experienced in effectively implementing SGBV policies and connected services might not be mere oversights. Instead, they can be viewed as integral to a broader pattern of neglect within the global health and development sphere. These constraints serve to maintain a power dynamic and diverts attention away from more equitable and effective alternatives, ultimately hindering progress toward a liberatory approach to global health and development. How can an industry that claims to advocate for a decolonial praxis, which seeks to overhaul existing systems, do so while neglecting fundamental necessities crucial for its efforts to be effective and sustainable? There is a contradiction between the industry's aspirations for decolonization and its current systemic neglect of basic resources necessary for progress. Kim [60] emphasizes the implicit ideological functions of global health and development, stating that the mere actions and operations of its institutions explicate that the functioning of this industry is precisely to “serve the hegemonic value of its time” ([60], p. 2). They go on to say how the concentration of resources and power for the privileged is a systemic feature, not a coincidence, therefore making it necessary for foreign aid to exist.

This compounding reality of resource scarcity within Global Majority countries against wealth and resource hoarding within the global health and development industrial complex is relevant for the provision of SGBV services, as it creates a formidable barrier to engaging and involving local communities in meaningful ways. When essential resources are constrained, the ability to establish trust, provide consistent support, and ensure timely responses to survivors is compromised. This, in turn, undermines efforts to sensitize and mobilize communities against sexual violence. The scarcity-driven environment leaves local communities feeling neglected and disempowered, impeding their active participation in the process. Consequently, global health and development not only perpetuates exploitative power dynamics but also significantly diminishes the feasibility of fostering genuine community involvement in combating sexual violence. This evidence illuminates the stark reality that the industry, despite its stated intentions, lacks the preparation or commitment required to enact the systemic changes necessary for an authentic decolonization process.

### Institutionalization of SGBV and broader hegemonic beliefs

Beyond the challenges posed by resource misallocation, stakeholders described how institutional and societal understandings of SGBV are shaped by dominant knowledge systems and exclusionary ideologies. When asked about the facets of their interventions that stakeholders took pride in, as well as the hurdles they encountered in their SGBV efforts, knowledge acquisition and data collection emerged as important themes.

The practice of tracking, storing, and sharing data on SGBV—long considered the gold standard for evidence-based policy and intervention design [27, 61, 62]—draws heavily from Western knowledge systems. While valuable, these systems can perpetuate intellectual colonialism, privileging what is quantifiable while sidelining effective, context-specific approaches rooted in local wisdom and traditions [63]. This datafication of SGBV oversimplifies its complex, sociopolitical nuances [64], potentially crowding out the very epistemologies needed for transformative change. While evidence underscores the crucial need to prioritize marginalized populations within SGBV efforts [65, 66], stakeholders noted the hegemonic beliefs that some marginalized groups conflict with Zambian (Christian) values which result in their silencing, hiding, and exclusion from these interventions. This hostile environment renders SGBV efforts incapable of being inclusive and participatory, which runs contrary to the essence of a decolonial praxis.

Moreover, these dominant knowledge systems and institutional values perpetuate institutionalized SGBV and hegemonic belief systems, not only in broader society but also within the institutions tasked with addressing SGBV and its root causes. While stakeholders often pointed to “bad culture” as the root of SGBV, this framing risks obscuring how institutions themselves reinforce exclusionary norms. Rather than addressing power asymmetries, many interventions mirror the very logics they claim to dismantle. When institutions adopt technocratic, individualized responses—often driven by donor preferences—they neglect the structural roots of violence and fail to ensure safety for those most at risk. This disconnect reveals a critical tension: how can institutions meaningfully engage in a decolonial praxis when their own frameworks, language, and actors remain embedded in the same hegemonic ideologies that sustain inequity?

A critical discourse analysis of key policies shaping the Anti-GBV Act [67] reveals deeply ingrained hegemonic discourses constraining structural transformation in Zambian SGBV interventions. This analysis prompts global health and development stakeholders to reassess the efficacy of these discourses and confront the structural factors they often ignore. However, critiquing policy discourse is necessary but insufficient, as these policies are created by individuals shaped by dominant social constructs. Without confronting institutional complicity, reckoning with one’s own entanglement in hegemonic systems, and redistributing power, institutional claims to decolonize remain rhetorical rather than transformative.

Unfortunately, this guidance toward introspection and accountability stands in direct conflict with the ethos of global health and development, particularly under neoliberalism. Esposito and Perez [68] argue, neoliberalism reduces all aspects of human life to economic terms—a logic that permeates the global health industry, favoring scale, efficiency, and market-driven empowerment over collective healing, historical reckoning, and structural change.

Focusing on individualistic solutions, like economic empowerment [69], obscures the structural nature of SGBV [67] and limits the potential for radical, community-centered transformation needed for a decolonial praxis. Recognizing that true empowerment extends beyond commodified remedies, the industry must embrace collective, community-driven strategies that challenge entrenched hierarchies. Without recentering the voices and safety of the most marginalized, reliance on foreign health and development paradigms persists, leaving little capacity to envision alternative futures. Consequently, attempts to implement decolonial roadmaps become hollow gestures, disconnected from the very shifts they claim to promote.

While this study is grounded in Zambia’s sociopolitical and institutional context, the systemic and operational barriers identified—constrained resources, carceral logics, policy incoherence, and donor-driven priorities—reflect broader dynamics observable across many Global Majority settings. These findings may resonate beyond Zambia, but we caution against viewing them as universally transferable. The tendency in global health and development to generalize or “scale up” approaches across diverse contexts often reproduces colonial logics by overlooking historical, political, and cultural specificity. Instead, we offer these findings as a grounded, situated contribution to rethinking what decolonization might demand.

## Conclusion: questioning institutional motivations

In this paper, in response to idealistic calls for ‘decolonization’, and ‘roadmaps’ to its implementation, we have offered a qualitative study of the feasibility of decolonization in the context of SGBV policy and interventions in Zambia. Our findings highlight that the oft-cited challenges facing SGBV work—including limited resources, poor coordination, prioritization of financial gains over addressing SGBV, misallocation of funds, entrenched social norms, institutionalization of SGBV, and marginalization of specific community groups—are not simply logistical or technical issues. Rather, they are systemic and operational manifestations of a global health and development paradigm that continues to reproduce colonial power relations. These conditions are fundamental obstacles that make decolonial praxis unworkable under current institutional arrangements. We argue that the global health and development industrial complex’s embrace of decolonial rhetoric often obscures its failure to address these structural barriers in meaningful ways. The assumption that adopting decolonial language equates to enacting decolonial change neglects the deep work of community empowerment and systemic transformation. Our findings show that the very barriers often viewed as implementation obstacles are, in fact, the core impediments to decolonial action — emphasizing the importance of not only aligning interventions with decolonial goals, but transforming the conditions that undermine them.

Considering how global health and development could embrace decolonization, it’s crucial to recognize the inherently political nature of this endeavor. The industry's pretense of apolitical intentions [70] not only undermines its capacity for genuine transformation but also perpetuates systemic issues by overlooking power dynamics, profit-driven motives, and hegemonic ideologies woven into the fabric of interventions and the ways data is collected and knowledge produced. This pretense of neutrality also obscures how positionalities—of institutions, funders, and practitioners—shape what is seen as valid, urgent, or possible within SGBV efforts. The existing structures have perpetuated carcerality, constrained resources, and reinforced dominant narratives, significantly impeding the industry’s readiness to adopt decolonial praxis. Genuine transformation demands an honest acknowledgment of the industry's political nature, a reckoning with its positionalities, and a conscientious commitment to reshaping power dynamics.

In the quest for transformation, one of the grassroots stakeholders profoundly encapsulated the actual community desires:

“What I want is a society where we feel free and safe knowing we will never experience violence again.” (Grassroots organization)

This statement underscores the true nature of empowerment in contrast to the institution’s individualized construction. It highlights that despite institutions promoting economic and educational empowerment as liberating [67, 69], marginalized groups still feel unsafe. If individuals do not feel safe in supposedly empowering spaces, vulnerability persists, hindering meaningful engagement. Therefore, prioritizing the safety and freedom of marginalized populations is foundational, and substantive, transformative change beyond policy rhetoric is necessary. It serves as a poignant reminder of the overarching goal: safety and security for all, requiring a collective commitment to address systemic and operational barriers hindering progress towards a decolonized approach in global health and development.

Authentic engagement in transformative decolonial praxis requires acknowledging and dismantling structural barriers, redistributing power and resources to create space where communities can define and lead efforts to build a world free from violence and inequity.

## Supplementary Information

Supplementary Material 1.

## Data Availability

The datasets generated and/or analyzed during the current study are not publicly available due to the sensitive nature of the data and the need to protect the confidentiality and privacy of the participants. Additionally, the data contain detailed personal narratives and potentially identifiable information that could compromise the anonymity of the participants. Therefore, to uphold ethical standards and ensure participant confidentiality, the raw interview data cannot be made publicly available.

## Abbreviations

Anti-GBV Act: Anti-Gender-Based Violence Act
GHDM-Africa: Global Health Decolonization Movement in Africa
HHCC: London School of Hygiene and Tropical Medicine’s Health in Humanitarian Crises Centre
IMF: International Monetary Fund
IPV: Intimate partner violence
MMD: Movement for Multiparty Democracy
NCDs: Non-communicable diseases
OSCs: One-stop centers
SADC: Southern Africa Development Community
SGBV: Sexual and gender-based violence
SRHR: Sexual and reproductive health and rights
STOP GBV: Stop Gender-Based Violence
VSU: Victim support units
