# Supplementary material for: “I think it is quite naive to think everybody’s goal is that”: how Zambian sexual violence stakeholder perspectives complicate global health roadmaps to ‘decolonization’
Source: BMC Health Serv Res. 2025 Oct 15;25:1364. doi: 10.1186/s12913-025-13188-5 (PMC12523078; doi:10.1186/s12913-025-13188-5)
Supplement: Supplementary file 1 — Supplementary Material 1. [file 12913_2025_13188_MOESM1_ESM.docx]

**Semi-Structured Interview Guide for Exploring SGBV Interventions in Zambia**

Nancy Nyutsem Breton

Mazuba Mushota-Mafwenko

Nancy Lwimba Mukupa

Interviewee’s role in international development

1. Could you please speak briefly on how you got involved in international development and how you came to be in your current role?
2. What does your current role entail and how does it support the overall mission of your project and/or organization?

Interviewee’s perspective on sexual and gender-based violence (SGBV) in Zambia

1. How do you understand the problem of SGBV, and how do you see it should or can be changed for the better? *(Does the interviewee see it as a feminist issue, a structural issue, a personal/individual responsibility issue, etc.? How do they view the responsibility of their organization, as well as other national/international organizations, in responding to SGBV?)*
   1. It is a big question, but why is SGBV such a problem in Zambia? What are the causes and issues?
   2. And how does your organisation fit in? In which bit of this system do you try to intervene?
   3. What are the changes you are trying to bring about?

Organizations’ mission and what they are trying to do in-country, particularly in SGBV

1. What role does your organization play in preventing and responding to gender-based violence in Zambia?
   1. Who are your target audience?
   2. How and Why are your efforts directed to them?
2. What have been the notable successes of your interventions?
   1. And what have you been most proud of, personally?
3. What challenges have you/your organization faced when trying to implement/evaluate work related to gender-based violence?
   1. How have you tried to overcome these challenges? OR What can be done to overcome these challenges OR What needs to be done to overcome these challenges?
4. How are successes and challenges of interventions measured?
   1. And beyond measurement, are there other ways that successes are appreciated by different groups? For example: by communities/beneficiaries/employees; through local celebrations / awards among different NGOs/policy makers; as success stories/examples in news reports, trainings, best practice reports, etc?
5. How are the lessons learned applied to improve your implementation of the interventions?

Policy perspective and involvement

1. What are key international and/or local policies that your organization utilizes in its work and how do these reinforce/support your work?
2. What are the policies designed to achieve? What changes do they try to bring about?
3. Could you please give any details on the international development programs or policies related to the SGBV that you have worked on that you are considerably proud of? *List them, and why they are proud of each.*
   1. (If the interviewee does not mention Zambia’s 2011 Anti-GBV Act): Are you aware of Zambia’s 2011 Anti-Gender Based Violence Act?
      - *(If yes):* What is your perception on if and how this policy has influenced SGBV interventions within your organization and the way that SGBV is addressed in Zambia as a whole?

Collaborating with local partners and population groups

1. Have you collaborated with Zambian government bodies, grassroots organizations, or local community leaders in SGBV interventions?
   1. If yes, to what extent do/did you collaborate with these? What does/did that look like?
   2. If no, why not?
   3. Are there any barriers or challenges in doing so? What are they?
2. Can you give an example on how your organization decides to work with key populations, and what that work may look like?
3. Can you think of any notable moments where your organization partnered with local NGOs for SGBV/sexual health interventions? If so, what did that partnership look like?
4. To what extent do you think/feel there is harmonised efforts amongst various stakeholders in responding to SGBV?
   1. *In what sense is there harmony/coordination/collaboration? In what sense is there* ***no*** *harmony/coordination/collaboration?*

Decolonising SGBV and other global health interventions

1. We hear growing concerns to 'decolonise' development and global health. Does this movement resonate with you? If so, how? If not, why not?
   1. Have you been involved in or heard of any such initiatives?
   2. What are the challenges and opportunities in this movement?
2. How do you see the field developing over the next 5-10 years?
   1. What are the existing priorities?
   2. What changes would you like to see come about? *Particularly in relationship with this call to decolonise.*
      - In terms of SGBV policy, practice, and the structures that they lie within?
      - In terms of perceptible changes to the lives of people and the communities they encompass?

Ending the interview

1. Is there anything else you would like to mention before we end the interview?
